# Supplementary material for: Different In Situ Immune Patterns between Primary Tumor and Lymph Node in Non-Small-Cell Lung Cancer: Potential Impact on Neoadjuvant Immunotherapy
Source: J Immunol Res. 2022 Apr 28;2022:8513747. doi: 10.1155/2022/8513747 (PMC9071859; doi:10.1155/2022/8513747)
Supplement: Supplementary Materials — Supplementary Table 1: densities and correlations of CD3+ and CD8+ lymphocyte in tumor center and invasive margin between primary tumor and metastatic lymph node. Supplementary Table 2: density and frequency of S-PD-L1-positive T cells in primary tumor and metastatic lymph node. Supplementary Table 3: densities of stromal CD3+, CD8+, and PD-L1-positive CD3+ lymphocytes in primary tumor and metastatic lymph nodes. Supplementary Figure 1: the tumor proportion score (TPS, %) was significantly correlated between primary tumors (PTs) and metastatic lymph nodes (mLNs) (r = 0.40, P = 0.016), but the combined positive score (CPS) was not (r = 0.14, P = 0.42). Supplementary Figure 2: forest plots showing pathological complete response in primary tumor versus complete nodal clearance (ypN0) following neoadjuvant immunotherapy (data extracted from five trials). Supplementary Material: case presentation. [file 8513747.f1.zip › Manuscript R1 highlighted.docx]

**Different *in situ* immune patterns between primary tumor and lymph node in non-small cell lung cancer: potential impact on neoadjuvant immunotherapy**

Zheng-Hao Ye^1,2^, Hao Long^1,2^, Ze-Rui Zhao^1,2*^

^1^. State Key Laboratory of Oncology in Southern China, Collaborative Innovation Center for Cancer Medicine, and Department of Thoracic Surgery, Sun Yat-Sen University Cancer Center, Guangzhou, China

^2^. Lung Cancer Research Center, Sun Yat-Sen University, Guangzhou, China

*****Corresponding author: Ze-Rui Zhao MD PhD, zhaozr@sysucc.org.cn, +86 87343261. Department of Thoracic Surgery, Sun Yat-Sen University Cancer Center, Guangzhou, China, 510060.

**Acknowledgement:** Part of the contents of this study was presented as poster in the 2020 World Conference on Lung Cancer.

**Abstract**

**Background** Neoadjuvant immunotherapy is promising for locally-advanced non-small cell lung cancer (NSCLC). The *in situ* immune patterns, as predictor of PD-1/PD-L1 blockade outcomes, of the primary tumor (PT) and metastatic lymph nodes (mLNs) is unknown.

**Methods** Multiplex immunofluorescence staining and multispectral imaging were used to evaluate the *in situ* immune patterns of T cells (CD3+) and cytotoxic T cells (CD8+) in terms of density, location (center of tumor [CT] and invasive margin [IM]), and the PD-L1 expression status of tumor cells and stromal T cells of paired PTs and mLNs in 38 stage-III NSCLCs.

**Results** The densities of T cells and cytotoxic T cells were correlated between PTs and mLNs at both CT and IM. Higher densities of stromal T cells (S-CD3+) at CT and both S-CD3+ and cytotoxic T cells (S-CD8+) at IM were observed in mLNs compared to PTs, while in tumor compartment, there were no differences in the densities of T cells (T-CD3+) or cytotoxic T cells (T-CD8+). Only the density of stromal PD-L1-positive T cells (S-PD-L1+CD3+) at CT was correlated between PTs and mLNs, while the densities and frequencies of S-PD-L1+CD3+ at CT and IM of mLNs were higher than PTs. Combine positive score discordance of PD-L1 between PTs and mLNs was greater than tumor proportion score.

**Conclusions** *In situ* immune patterns of T cells and cytotoxic T cells were different between PTs and mLNs in NSCLC. ~~Higher densities of S-CD3+ and S-PD-L1+CD3+ in mLNs may result in better immune-mediated response to neoadjuvant immunotherapy.~~ The heterogeneity of the *in situ* immune patterns may result in different immune-mediated responses to neoadjuvant immunotherapy in PT and mLNs.

**Keywords:** Non-small cell lung cancer; Primary tumor; Lymph node; Immune pattern

**INTRODUCTION**

Lung cancer is the leading cause of cancer-related death worldwide [1]. The discovery of programmed cell death 1 (PD-1) and its ligand (PD-L1) improved the management of advanced-stage lung cancer by PD-1/PD-L1 blockade [2-4]. To date, the most widely used biomarker for immunotherapy is PD-L1 expression on tumor or immune cells, although its accuracy for predicting the response to immunotherapy is insufficient [5, 6]. The tumor immune microenvironment (TIME), particularly the *in situ* immune pattern or contexture, defined as the type, functional orientation, density, and location of adaptive immune cells within distinct tumor regions, demonstrates the effectiveness of host immune response to tumor or tumor-host interaction and may influence the efficacy of immunotherapy [7-9]. Moreover, this phenomenon exists not only in primary tumors (PTs) but also in metastatic lesions. For example, patients with liver metastases of colorectal cancer (CRC) with higher density of tumor infiltrating lymphocytes (TILs) at the invasive margin (IM) have prolonged progression-free survival with chemotherapy [10].

Compared to the TNM staging system, a scoring system based on the immune contexture and incorporating the host immune response, called immunoscore (IS) [11], is a useful prognostic factor for solid tumors, including non-small cell lung cancer (NSCLC) [12-15]. Therefore, an international task force was established to incorporate the IS into the traditional classification, designated TNM-immune, to improve the prediction of the outcomes of malignancies [16, 17].

According to the IS task force, CD3 and CD8 in two regions (center of tumor [CT] and IM), are used for validating the IS [16]. As the immune response to cancer depends on T cells that recognize cancer-associated antigens [18], CD3, as pan-T-cell marker, has a positive prognostic impact in NSCLC patients. In addition, patients with a high density of CD3+ TILs have a favorable prognosis [19, 20]. CD8, a marker of cytotoxic T cells, which play a pivotal role in antitumor immune responses, is also a prognostic factor for NSCLC [17]. Recently, stromal CD8+ TILs at IM region were proved to be an independent prognostic factor and IS indicator for NSCLC [21].

~~Because the~~ *~~in situ~~* ~~immune patterns of PTs or metastases have prognostic and therapeutic implications, we hypothesized that the immune patterns of PT and~~ ~~metastatic lymph nodes (~~~~mLNs) of stage III NSCLC could have potential impact on their response to neoadjuvant immunotherapy. In this study, multiplex immunofluorescence and multispectral imaging were used to evaluate the~~ *~~in situ~~* ~~immune patterns of T cells (CD3+) and cytotoxic T cells (CD8+) in terms of their density and location. A preprint has previously been published [22] and we also analyzed PD-L1 expression on tumor cells and stromal T cells to see if there is difference of~~ *~~in situ~~* ~~immune patterns between the PTs and mLNs of NSCLC.~~

Given that the *in situ* immune patterns of either PTs and metastases had prognostic and therapeutic implications, we used multiplex immunofluorescence and multispectral imaging to evaluate the *in situ* immune patterns of T cells (CD3+) and cytotoxic T cells (CD8+) in terms of their densities and locations to explore if there was a difference of *in situ* immune patterns between the PTs and mLNs of NSCLC in this study. A preprint has previously been published [22], analysis of PD-L1 expression on tumor cells and stromal T cells was conducted as well.

**MATERIALS and METHODS**

**Patients and Specimens**

~~Patients who underwent lobectomy/pneumonectomy with lymphadenectomy with curative intent from 2012 to 2016 and were pathologically diagnosed with lung~~ ~~adenocarcinoma (~~~~ADC) or~~ ~~squamous cell carcinoma (~~~~SCC) with mediastinal lymph node metastases (N2) were reviewed. Patients with an~~ *~~EGFR~~* ~~mutation,~~ *~~EML4-ALK~~* ~~translocation, or insufficient formalin-fixed, paraffin-embedded tissue for PT or mediastinal mLNs were excluded.~~ During the initial screening phase, treatment-naive patients who underwent lobectomy/pneumonectomy with lymphadenectomy with curative intent from 2012 to 2016 were screened and retrieved from the electronic medical record database. Then patients who were pathologically diagnosed with lung adenocarcinoma (ADC) or squamous cell carcinoma (SCC) with mediastinal lymph node metastases (N2) were identified using the electronic pathology database. Patients with EGFR mutation and EML4-ALK translocation were excluded in the meantime. We reviewed the pathological report in detail to identify the metastatic lymph node with the largest diameter in N2 stations. Finally, the paraffin sections of both the PTs and mLNs were reviewed by two pathologists. Only samples with CT and IM at both PTs and mLNs were selected and sent for final analyses. Demographic data, smoking status, surgical procedure, and pathologic information were retrieved from medical records. Immune-related pathologic response criteria proposed by Cottrell, T. R *et al.* were used to evaluate PTs and mLNs following immunotherapy [23]. In their system, the total tumor bed is defined by residual viable tumor + necrosis + regression bed. The total tumor bed is estimated to calculate the percentage of immune-related residual viable tumor (viable tumor area/total tumor bed area * 100). This study was conducted in accordance with the amended Declaration of Helsinki. The institutional review board approved the study protocol, and written consent for tissue analyses were obtained from all patients preoperatively.

**Multiplex Immunofluorescence Staining/ Multispectral Imaging**

Multiplexed immunoﬂuorescence staining was performed according to the tyramide signal amplification immunostaining method to visualize the expression of CD3, CD8, PD-L1, and cytokeratin on sequential 4 μm sections of formalin-fixed, paraffin-embedded tissue of PTs and mLNs using the PANOVUE 7-plex IHC kit, (Panovue, Beijing, China) [24]. Staining for CD3, CD8, PD-L1, and cytokeratin was performed sequentially using primary antibodies to detect epithelial tumor cells (type II cytokeratin, 1:400; clone BP6058, Biolynx), T lymphocytes (CD3, 1:200; clone SP7, Abcam), cytotoxic T cells (CD8A, 1:200; clone C8/144B, CST), and PD-L1 (clone SP142, 1:400; Abcam) followed by incubation with the horseradish peroxidase-conjugated secondary antibody and signal amplification using tyramide-conjugated fluorophores. Nuclei were stained with 4′-6′-diamidino-2-phenylindole (DAPI; Sigma-Aldrich) after all human antigens had been labeled. Human tonsil tissues were used with and without primary antibodies as positive and negative (autofluorescence) controls, respectively.

For multispectral imaging, stained slides were scanned and imaged using the Polaris System (PerkinElmer, Waltham, MA) to capture the fluorescent spectrum at 20 nm wavelength intervals from 420 to 720 nm, which were combined to build a single stack image. To extract the autofluorescence spectrum of tissue and each fluorescein, unstained and single-stained images were used. For multispectral unmixing, a spectral library was established using the extracted images and Inform 2.4 image analysis software (PerkinElmer, Waltham, MA). Using the spectral library, we generated reconstructed images of sections without autofluorescence. The multispectral images containing PD-L1, CD3, CD8A, and cytokeratin were analyzed by Inform 2.4 image-analysis software using the tissue finder tool to segment the images into the tumor compartment (presence of cytokeratin [CK] positivity), stroma compartment (absence of cytokeratin positivity), and blank tissue compartment (absence of cells). Individual cells (DAPI+) were identified by adaptive segmentation algorithms to identify co-localization of cell populations and compartments, which were labeled as follows: tumor cells (CK+), cells expressing PD-L1 (PD-L1+), tumor cells expressing PD-L1 (PD-L1+CK+), T cells (CD3+), cytotoxic T cells (CD8+), T cells expressing PD-L1 (PD-L1+CD3+), and cytotoxic T cells expressing PD-L1 (PD-L1+CD8+). Analyses using PanoScore 1.0 software (Panovue, Beijing, China) generated a cell-by-cell identification report of cell populations in the three compartments, including their density (number of cells/mm^2^).

**Assessing in situ Immune Pattern**

PTs and matched mLNs sections at low magnification (×10) were scanned to identify CT and IM using the Polaris System (PerkinElmer, Waltham, MA) (Fig.1A), as described [16, 21]. The same numbers of fields of view (FOVs) at high resolution (×20) were taken in each region, if possible. The numbers of FOVs were based on the size of viable tumor tissue, if the tumor tissue was too small, representative FOV would be taken and categorized as CT (Fig. 1B). For tumor tissue that scattered and could not be divided into CT and IM regions, the FOV of each tumor area was collected and categorized as IM. The same numbers of FOVs at high resolution (×20) were taken in each region, if possible (Fig. 1). To compare the immune contexture of T cells (CD3+) and cytotoxic T cells (CD8+), the densities (cells/mm^2^) of each between PTs and mLNs were sorted in descending order and matched in the same patient.

**Assessment of** **PD-L1 Expression**

Because PD-L1 expression on tumor-infiltrating T cells reflects an ongoing immune response and has an intrinsically favorable prognostic impact [7], and patients with higher density and frequency of stromal PD-L1-positive regulatory T cells had a better response to immunotherapy [25], we investigated whether the densities or frequencies of stomal tumor-infiltrating T cells in CT and IM regions was correlated between PTs and mLNs. The highest density and frequency of stromal PD-L1-positive T cells (S-PD-L1+CD3+) in each region were analyzed. The frequency was calculated as the number of PD-L1-positive T cells divided by the number of CD3+ cells in the stroma compartment multiplied by 100.

Additionally, views of CT scans with a minimum of 100 tumor cells were eligible for analyses of PD-L1 expression in PTs and mLNs [26]. PD-L1 expression on tumor cells was evaluated using the tumor proportion score (TPS), which was the number of PD-L1-stained tumor cells (PD-L1+CK+) divided by the total number of viable tumor cells (CK+) multiplied by 100 in the tumor compartment. PD-L1-stained cells were evaluated by calculating the combined positive score (CPS) in the total compartment, which was the number of PD-L1-stained cells (PD-L1+) divided by the total number of viable tumor cells (CK+) multiplied by 100. The TPS is considered positive at ≥ 1%, and the CPS at > 1.

**Statistical Analysis**

The Spearman rank correlation test and Wilcoxon signed-rank test (paired, nonparametric) were used to evaluate the densities of TILs in CT and IM regions of PTs and mLNs. For analyses of PD-L1 expression between PTs and mLNs, Cohen’s κ coefficient of agreement was used to classify the level of concordance as poor (κ = 0.00), slight (κ = 0.00–0.20), fair (κ = 0.21–0.40), moderate (κ = 0.41–0.60), substantial (κ = 0.61–0.80), or almost perfect (κ = 0.81–1.00) [27]. Data from published trials were collected and the pooled analysis was performed to demonstrate the response of PT and mLN in patients with NSCLC following neoadjuvant immunotherapy using Review Manager 5.3 (RevMan; Cochrane Collaboration). Pathological complete response of the PT and node downstaging were recoded into dichotomous variables, which were further compared by the odds ratio. All tests were two-sided and a P value < 0.05 were considered statistically significant. Statistical analyses and plots were conducted using R (3.5.3 GUI 1.70 El Capitan build [7632]; [www.R-project.org](http://www.R-project.org)).

**RESULTS**

**Clinicopathological Characteristics**

Thirty-eight patients were enrolled and their clinicopathologic characteristics are shown in Table 1. All patients were pathologically staged as III, and the median age was 55.50 (range 34–72) years.

**Different in situ Immune Pattern of T Cells and Cytotoxic T Cells between PTs and mLNs**

Given that no significant difference was found between SCC and ADC regarding *in situ* immune pattern in this study, analyses were conducted for the entire cohort. In the CT and IM tumor compartments, the densities of T-CD3+ and T-CD8+ cells were not significantly different but were significantly correlated between PTs and mLNs (*P* < 0.0001). Therefore, the *in situ* immune patterns of T-CD3+ and T-CD8+ were reproduced from PTs to mLNs during metastasis in both CT and IM regions. The density of S-CD3+ cells was significantly higher in mLNs than in PTs for both CT and IM regions (*P* < 0.0001), whereas that of S-CD8+ cells was so only in the IM (*P* < 0.0001). The densities of stromal T cells and cytotoxic T cells were also significantly correlated between PTs and mLNs (*P* < 0.0001) (Fig. 2; Supplementary Table 1).

**Density of Stromal PD-L1-positive T cells in CT is Correlated between PTs and mLNs**

The densities of stromal PD-L1-positive T cells were significantly correlated between PTs and mLNs at CT (*P* = 0.020) (Fig. 3A). However, in IM region, it was only a tendency (*P*= 0.051) (Fig. 3B). The frequencies of stromal PD-L1-positive T cells were not significantly correlated in CT and IM regions. Irrespective of location, the densities and frequencies of stromal PD-L1-positive T cells were significantly higher in mLNs than in PTs (*P* < 0.05) (Fig. 3; Supplementary Table 2).

**CPS is More Discrepant than TPS between PTs and mLNs**

Three cases were excluded from analyses of PD-L1 expression due to the absence of scans of PTs or mLNs or <100 tumor cells per FOV, hence ineligible for TPS and CPS calculation. Of the other 35 cases, the concordance rate of the TPS distribution of PT and mLN in a single participant was 66% (23/35) (κ = 0.302, fair agreement). The TPS (%) of the PTs and mLNs were significantly correlated (Supplementary Fig. 1A). Besides, the concordance rate between PTs and mLNs of CPS was 60% (21/35) (κ = 0.116, slight agreement). There were no significant correlations of CPS between PTs and mLNs (Supplementary Fig. 1B).

**~~Better Immune-mediated Response of mLN than PT after Neoadjuvant Immunotherapy~~**

**Different Immune-mediated Response in mLN and PT after Neoadjuvant Immunotherapy**

In a patient with stage IIIa (N2) SCC who received three-cycles of neoadjuvant pembrolizumab + chemotherapy (Supplementary Material), the percentage of immune-related residual viable tumor was 60% in PT and 0% in mLNs following complete resection. Both PT and mLNs showed immune-mediated responses such as proliferative fibrosis, neovascularization and necrosis. However, foamy macrophages and cholesterol clefts were found exclusively in mLN. Interestingly, the densities of S-CD3+ in CT and IM regions as well as S-CD8+ in CT region in PT were significantly lower than in mLNs (*P* = 0.0016). The density of S-PD-L1+CD3+ was only significantly higher in mLN than in PT at CT (*P* = 0.047). No difference was found in the densities of S-CD8+ and S-PD-L1+CD3+ between PT in IM region and mLNs, which may be due to higher densities of these cells at IM than at CT in PT of this patient (*P* = 0.016) (Fig. 4, Supplementary Table 3).

~~Data extracting from five phase-II neoadjuvant immunotherapy trials showed that complete nodal clearance was more likely to occur than pathological complete response in the PT in NSCLC patients following neoadjuvant immunotherapy (odds ratio=3.56; 95% confidence interval 2.14-5.92;~~ *~~P~~* ~~< 0.001; Supplementary Fig. 2) [28-32].~~

Data extracted from five neoadjuvant immunotherapy trials also showed that there were different pathological responses between mLNs and PTs. Complete nodal clearance (ypN0) was more likely to occur than pathological complete response in the PT (ypT0) in NSCLC patients following neoadjuvant immunotherapy (odds ratio=3.56; 95% confidence interval 2.14-5.92; *P* < 0.001; Supplementary Fig. 2) [28-32].

**DISCUSSION**

We conducted a comparative study of the *in situ* immune patterns of PTs and mLNs of NSCLC patients regarding the spatial distributions and densities of CD3+ and CD8+ TILs and the PD-L1 expression statuses of tumor cells and stromal CD3+ TILs. Results showed that the densities of T-CD3+ and T-CD8+ cells were not significantly different yet significantly correlated between PTs and mLNs in the tumor compartment, i.e., the *in situ* immune patterns of T-CD3+ and T-CD8+ were homogenous. Although the densities of S-CD3+ and S-CD8+ were significantly correlated, their *in situ* immune patterns were heterogeneous, with a higher density of the former at CT and IM and a higher density of the latter at IM in mLNs.

Interestingly, Remark *et al*. reported similar results for PTs and lung metastases in CRC and renal cell carcinoma (RCC) patients [33]. In their work, the densities of CD8+, DC-LAMP+, and NKp46+ TILs (markers of cytotoxic T cells, mature dendritic cells, and natural killer cells, respectively) were positively correlated between primary CRC and RCC tumors and corresponding lung metastases (r = 0.656 to 0.693 for CRC and 0.547 to 0.817 for RCC). However, the densities of CD8+ T cells of CRC-PT and DC-LAMP+ TILs of RCC-PTs were significantly higher than in lung metastases. It is possible that the *in situ* immune pattern reproduced from PTs to metastases occurs not only in CRC and RCC but also in NSCLC, and the reproducibility was shown in both CT and IM regions in our study. This supports the theory that there is imprinting of the TIME by tumor cells as the immune cells are “educated” by the immune contexture of PT and recalled at metastatic sites [33].

Patients with a pre-existing T-cell-infiltrated tumor microenvironment are more likely to respond to immunotherapy, as the checkpoint inhibitors can enhance the pre-existing immune response and may induce new T cells-mediated-immune responses [7]. Melanoma with a higher density of CD8+ cells in the IM region had a better response to the immunotherapy and exhibited a parallel increase in CD8+ cells in both the IM and CT regions [34]. Wu *et al*. reported that patients with a higher frequencies and densities of stromal PD-L1-positive regulatory T cells (CD25+ CD4+) and PD-1-positive CD8+ T cells have a better response to immunotherapy [25]. In this study, we investigated the PD-L1 expression status of stromal tumor-infiltrating T cells in terms of density and location in PTs and mLNs. The densities of S-PD-L1+CD3+ at CT were significantly correlated between PTs and mLNs. However, only a marginally significant correlation was found at IM. The densities and frequencies of S-PD-L1+CD3+ were higher in mLNs than in PTs in both regions. These results suggest that PD-L1 expression of stromal tumor-infiltrating T cells differs between PTs and mLNs, and the ongoing immune response is stronger in mLNs than in PTs in NSCLC patients.

Preclinical study had proved that tumor-draining, but not nondraining, lymph nodes served to accumulate T cells required for checkpoint blockade therapy to the PT [35]. To assess whether the finding in this study affects the response to neoadjuvant immunotherapy, a stage IIIa SCC underwent neoadjuvant chemo-immunotherapy was investigated. Interestingly, the percentage of immune-related residual viable tumor of PT and mLNs were 60% and 0%. The discrepant immune-related pathologic response between PT and mLNs may result from a more intense immune-response in mLNs than in PT at CT region as the densities of S-CD3+, S-CD8+ and S-PD-L1+CD3+ were higher in mLNs. Besides, the densities of S-CD8+ and S-PD-L1+CD3+ in IM region of PT were ~~no~~ not different ~~than~~ from mLNs. These results suggest that the immune-response of IM in PT ~~is~~ was similar to mLNs and stronger than of CT in PT, and IM region may serve as a frontline during the anti-tumor response, which results in immune-mediated features like the regression bed [28]. According to the study of Ling et al., the PT and mLN show various immune phenotypes following neoadjuvant immune checkpoint inhibitor monotherapy [36]. Considering that the pathological response of NSCLC after neoadjuvant immunochemotherapy is generally greater than that of anti-PD-1 monotherapy, the change of immune pattern by different neoadjuvant regimens remains to be investigated.

Several studies have compared the PD-L1 expression status of tumor cells between PTs and mLNs in NSCLC patients [27, 37, 38]. Uruga *et al*. reported that the discrepancy rate between PTs and mLNs was 9.4% to 15% [38]. In Kim *et al*., the concordance rate between PTs and metastatic sites (83.2% were regional lymph nodes) was 80.1% (κ = 0.492) in ADC patients [27]. Sakakibara *et al*. suggested that the expression levels of PD-L1 in tumor cells were significantly correlated between PTs and resected mLNs (r = 0.49, *P* < 0.001) [37]. In our study, the concordance rate of PD-L1 positivity between PTs and mLNs was 66% (κ = 0.302), and the TPS (%) between PTs and mLNs were significantly correlated.

Conversely, few studies have compared the CPS between PTs and mLNs. CPS which integrates all PD-L1 expressing cells (tumor cells, lymphocytes, and macrophages) is a prognostic indicator in patients treated with pembrolizumab [26, 39]. In this study, the concordance rate of CPS status was 60% (κ = 0.116) and was not correlated between PTs and mLNs. More heterogeneity in CPS than the TPS between PTs and mLNs may be explained by the composition of cells other than tumor cells differs dramatically between PTs and mLNs. Given that PD-L1 expression in stromal tumor-infiltrating T cells was higher in mLNs than in PTs, we postulated that the PD-L1 expression status of lymphocytes and macrophages would be heterogeneous, resulting in the discrepancy of CPS. Nodal status following induction therapy in NSCLCs affects the final pathological stage and thus determines the potential adjuvant therapeutic strategy after operation. As indicated by a recent published research, nodal disease status following neoadjuvant chemotherapy is a key determinant of survival among patients with major pathological response within the primary tumor [40]. It is plausible that the findings of the current study may have impact ~~in~~ on patients who undergo neoadjuvant immunotherapy.

Interestingly, the pathological response following neoadjuvant immunotherapy had been validated in several phase II trials, with major pathological response rates ranging from 18% to 83% [28-32]. The ~~extracting~~ extracted data from these trials indicated that for patients who achieved pathological complete response in their PTs, there ~~is~~ was a high tendency that the mLNs may experience downstaging to ypN0 following neoadjuvant immunotherapy. This, may to some extent, support the findings of the current study that there could be a more extensive response in the lymph nodes following immunotherapy.

The current study had several limitations. First, only patients with stage III (N2) disease without an *EGFR* mutation or *EML4-ALK* translocation were included, as such patients would likely derive the greatest benefit from neoadjuvant immunotherapy. Therefore, the results may not be generalizable to other populations of NSCLC patients. Second, ~~due to the small sample size,~~ potential bias due to the small sample size did exist in this study, and we could not assess whether the diverse *in situ* immune patterns between PTs and mLNs had a prognostic impact on patients following surgery. Third, though the results from the pooled analysis of published trials were used to indicate a potentially higher response rate of mLN following induction immunotherapy compared with PT, only one case ~~is~~ was depicted in this study to demonstrate the associated microenvironment. Whether the different immune-mediated responses in PT and mLN observed in this patient were related to the heterogeneity of the *in situ* immune patterns still needed further investigation for lacking the evaluation of preoperative samples of PT and mLNs in terms of baseline PD-L1 expression and TILs status. Last but not the least, the TILs assessed were limited to only two markers (CD3 and CD8). To evaluate *in situ* immune patterns more comprehensively, other markers such as CD4 (helper T cell marker), CD45RO (memory T cell), CD68 (macrophage), and FOXP3 (regulatory T cell) should be investigated.

**CONCLUSION**

Different *in situ* immune patterns of S-CD3+ and S-CD8+ as well as PD-L1 expression status of stromal T cells may result in different immune-related pathologic response between PTs and mLNs in NSCLC patients. Further studies are needed to clarify whether these phenomena impact the treatment and prognosis of NSCLC, particularly in the setting of neoadjuvant immunotherapy.

**Declarations**

**Funding:** This work was partly supported by the National Natural Science Foundation of China Youth Science Fund Project [grant number 82002407]. The funding body did not involve in the design of the study and collection, analysis, and interpretation of data and in writing the manuscript.

**Conflicts of interest statement:** The authors declare that they have no competing interests.

**Availability of data and material:** The datasets used and/or analyzed during the current study are available from the corresponding author on reasonable request.

**Author contributions:** (I) Conception and design: Zheng-Hao Ye, Ze-Rui Zhao; (II) Administrative support: Hao Long; (III) Provision of study materials or patients: Zheng-Hao Ye, Hao Long; (IV) Collection and assembly of data: Zheng-Hao Ye; (V) Data analysis and interpretation: : Zheng-Hao Ye; (VI) Manuscript writing: All authors; (VII) Final approval of manuscript: All authors.

**Ethical approval:** This study was conducted in accordance with the amended Declaration of Helsinki (as revised in 2013). The institutional review board approved the study protocol (SYSUCC B2019-116-01), and written consent for tissue analyses were obtained from all patients preoperatively.

**Consent for publication:** Not Applicable

**References**

[1]. Siegel RL, Miller KD, Jemal A. Cancer statistics, 2019. CA Cancer J Clin. 2019;69:7-34.

[2]. Mok TSK, Wu YL, Kudaba I, Kowalski DM, Cho BC, Turna HZ, et al. Pembrolizumab versus chemotherapy for previously untreated, PD-L1-expressing, locally advanced or metastatic non-small-cell lung cancer (KEYNOTE-042): a randomised, open-label, controlled, phase 3 trial. Lancet. 2019;393:1819-30.

[3]. Borghaei H, Paz-Ares L, Horn L, Spigel DR, Steins M, Ready NE, et al. Nivolumab versus Docetaxel in Advanced Nonsquamous Non-Small-Cell Lung Cancer. N Engl J Med. 2015;373:1627-39.

[4]. Socinski MA, Jotte RM, Cappuzzo F, Orlandi F, Stroyakovaskiy D, Nogami N, et al. Atezolizumab for First-Line Treatment of Metastatic Nonsquamous NSCLC. N Engl J Med. 2018;378:2288-301.

[5]. Brahmer J, Reckamp KL, Baas P, Crinò L, Eberhardt WE, Poddubskaya E, et al. Nivolumab versus Docetaxel in Advanced Squamous-Cell Non-Small-Cell Lung Cancer. N Engl J Med. 2015;373:123-35.

[6]. Rittmeyer A, Barlesi F, Waterkamp D, Park K, Ciardiello F, Pawel JV, et al. Atezolizumab versus docetaxel in patients with previously treated non-small-cell lung cancer (OAK): a phase 3, open-label, multicentre randomised controlled trial. Lancet. 2017;389:255-65.

[7]. Fridman WH, Zitvogel L, Sautes-Fridman C, Kroemer G. The immune contexture in cancer prognosis and treatment. Nat Rev Clin Oncol. 2017;14:717-34.

[8]. Fridman WH, Pages F, Sautes-Fridman C, Galon J. The immune contexture in human tumours: impact on clinical outcome. Nat Rev Cancer. 2012;12:298-306.

[9]. Angell H, Galon J. From the immune contexture to the Immunoscore: the role of prognostic and predictive immune markers in cancer. Curr Opin Immunol. 2013;25:261-7.

[10]. Halama N, Michel S, Kloor M, Zoernig I, Benner A, Spille A, et al. Localization and density of immune cells in the invasive margin of human colorectal cancer liver metastases are prognostic for response to chemotherapy. Cancer Res. 2011;71:5670-7.

[11]. Galon J, Pages F, Marincola FM, Thurin M, Trinchieri G, Fox BA, et al. The immune score as a new possible approach for the classification of cancer. J Transl Med. 2012;10:1.

[12]. Pages F, Kirilovsky A, Mlecnik B, Asslaber M, Tosolini M, Bindea G, et al. In situ cytotoxic and memory T cells predict outcome in patients with early-stage colorectal cancer. J Clin Oncol. 2019;27:5944-51.

[13]. Paulsen EE, Kilvaer T, Khanehkenari MR, Maurseth RJ, Al-Saad S, Hald SM, et al. CD45RO(+) Memory T Lymphocytes--a Candidate Marker for TNM-Immunoscore in Squamous Non-Small Cell Lung Cancer. Neoplasia. 2015;17:839-48.

[14]. Wang M. ImmunoScore predicts gastric cancer postsurgical outcome. Lancet Oncol. 2017;18:e68.

[15]. Kwak Y, Koh J, Kim DW, Kang SB, Kim WH, Lee HS. Immunoscore encompassing CD3+ and CD8+ T cell densities in distant metastasis is a robust prognostic marker for advanced colorectal cancer. Oncotarget. 2016;7:81778-90.

[16]. Galon J, Pages F, Marincola FM, Angell HK, Thurin M, Lugli A, et al. Cancer classification using the Immunoscore: a worldwide task force. J Transl Med. 2012;10:205.

[17]. Donnem T, Kilvaer TK, Andersen S, Richardsen E, Paulsen EE, Hald SM, et al. Strategies for clinical implementation of TNM-Immunoscore in resected nonsmall-cell lung cancer. Ann Oncol. 2016;27:225-32.

[18]. Chen DS, Mellman I. Elements of cancer immunity and the cancer-immune set point. Nature. 2017;541:321-30.

[19]. Schalper KA, Brown J, Carvajal-Hausdorf D, McLaughlin J, Velcheti V, Syrigos KN, et al. Objective measurement and clinical significance of TILs in non-small cell lung cancer. J Natl Cancer Inst. 2015;107:dju435.

[20]. Kayser G, Schulte-Uentrop L, Sienel W, Werner M, Fisch P, Passlick B, et al. Stromal CD4/CD25 positive T-cells are a strong and independent prognostic factor in non-small cell lung cancer patients, especially with adenocarcinomas. Lung cancer. 2012;76:445-51.

[21]. Donnem T, Hald SM, Paulsen EE, Richardsen E, Al-Saad S, Kilvaer TK, et al. Stromal CD8+ T-cell Density-A Promising Supplement to TNM Staging in Non-Small Cell Lung Cancer. Clin Cancer Res. 2015;21:2635-43.

[22]. Ye ZH, Zhao ZR. Different In Situ Immune Patterns Between Primary Tumor And Lymph Node In Non-Small Cell Lung Cancer: Potential Impact On Neoadjuvant Immunotherapy, 31 August 2021, PREPRINT (Version 1) available at Research Square [https://doi.org/10.21203/rs.3.rs-770110/v1].

[23]. Cottrell TR, Thompson ED, Forde PM, Stein JE, Duffield AS, Anagnostou V, et al. Pathologic features of response to neoadjuvant anti-PD-1 in resected non-small-cell lung carcinoma: a proposal for quantitative immune-related pathologic response criteria (irPRC). Ann Oncol. 2018;29:1853-60.

[24]. Stack EC, Wang C, Roman KA, Hoyt CC. Multiplexed immunohistochemistry, imaging, and quantitation: a review, with an assessment of Tyramide signal amplification, multispectral imaging and multiplex analysis. Methods. 2014;70:46-58.

[25]. Wu SP, Liao RQ, Tu HY, Wang WJ, Dong ZY, Huang SM, et al. Stromal PD-L1-Positive Regulatory T cells and PD-1-Positive CD8-Positive T cells Define the Response of Different Subsets of Non-Small Cell Lung Cancer to PD-1/PD-L1 Blockade Immunotherapy. J Thorac Oncol. 2018;13:521-32.

[26]. Kulangara K, Zhang N, Corigliano E, Guerrero L, Waldroup S, Jaiswal D, et al. Clinical Utility of the Combined Positive Score for Programmed Death Ligand-1 Expression and the Approval of Pembrolizumab for Treatment of Gastric Cancer. Arch Pathol Lab Med. 2019;143:330-7.

[27]. Kim S, Koh J, Kwon D, Keam B, Go H, Kim YA, et al. Comparative analysis of PD-L1 expression between primary and metastatic pulmonary adenocarcinomas. Eur J Cancer. 2017;75:141-9.

[28]. Forde PM, Chaft JE, Smith KN, Anagnostou V, Cottrell TR, Hellmann MD, et al. Neoadjuvant PD-1 Blockade in Resectable Lung Cancer. N Engl J Med. 2018;378:1976-86.

[29]. Gao S, Li N, Gao S, Xue Q, Ying J, Wang S, et al. Neoadjuvant PD-1 inhibitor (Sintilimab) in NSCLC. J Thorac Oncol. 2020;15:816-26.

[30]. Provencio M, Nadal E, Insa A, García-Campelo M.R, Casal-Rubio J, Dómine M, et al. Neoadjuvant chemotherapy and nivolumab in resectable non-small-cell lung cancer (NADIM): an open-label, multicentre, single-arm, phase 2 trial. Lancet Oncol. 2020;21:1413-22.

[31]. Rothschild S, Zippelius A, Eboulet E.I, Savic S, Betticher D.C, Bettini A, et al. SAKK 16/14: Anti-PD-L1 antibody durvalumab in addition to neoadjuvant chemotherapy in patients with stage IIIA(N2) non-small cell lung cancer (NSCLC)—A multicenter single-arm phase II trial. J Clin Oncol 2020;38:s9016.

[32]. Shu C.A, Gainor J.F, Awad M.M, Chiuzan C, Grigg C.M, Pabani A, et al. Neoadjuvant atezolizumab and chemotherapy in patients with resectable non-small-cell lung cancer: an open-label, multicentre, single-arm, phase 2 trial. Lancet Oncol. 2020;21:786-95.

[33]. Remark R, Alifano M, Cremer I, Lupo A, Dieu-Nosjean MC, Riquet M, et al. Characteristics and clinical impacts of the immune environments in colorectal and renal cell carcinoma lung metastases: influence of tumor origin. Clin Cancer Res. 2013;19:4079-91.

[34]. Tumeh PC, Harview CL, Yearley JH, Shintaku IP, Taylor EJ, Robert L, et al. PD-1 blockade induces responses by inhibiting adaptive immune resistance. Nature. 2014;515:568-71.

[35]. Fransen MF, Schoonderwoerd M, Knopf P, Camps MG, Hawinkels LJ, Kneilling M, et al. Tumor-draining lymph nodes are pivotal in PD-1/PD-L1 checkpoint therapy. JCI Insight. 2018;3:e124507.

[36]. Ling Y, Li N, Li L, Guo C, Wei J, Yuan P, et al. Different pathologic responses to neoadjuvant anti-PD-1 in primary squamous lung cancer and regional lymph nodes. NPJ Precis Oncol. 2020;4:32.

[37]. Sakakibara R, Inamura K, Tambo Y, Ninomiya H, Kitazono S, Yanagitani N, et al. EBUS-TBNA as a Promising Method for the Evaluation of Tumor PD-L1 Expression in Lung Cancer. Clin Lung Cancer. 2017;18:527-34.

[38]. Uruga H, Bozkurtlar E, Huynh TG, Muzikansky A, Goto Y, Gomez-Caraballo M, et al. Programmed Cell Death Ligand (PD-L1) Expression in Stage II and III Lung Adenocarcinomas and Nodal Metastases. J Thorac Oncol. 2017;12:458-66.

[39]. Bang YJ, Kang YK, Catenacci DV, Muro K, Fuchs CS, Geva R, et al. Pembrolizumab alone or in combination with chemotherapy as first-line therapy for patients with advanced gastric or gastroesophageal junction adenocarcinoma: results from the phase II nonrandomized KEYNOTE-059 study. Gastric Cancer. 2019;22:828-37.

[40]. Corsini EM, Weissferdt A, Pataer A, Zhou N, Antonoff MB, Hofstetter WL, et al. Pathological nodal disease defines survival outcomes in patients with lung cancer with tumour major pathological response following neoadjuvant chemotherapy. Eur J Cardiothorac Surg. 2021;59:100-8.

**Table 1**. Clinicopathological Characteristics

| Characteristics | Category | n (%) |
| --- | --- | --- |
| Age | ≤60 | 25 (66) |
|  | >60 | 13(34) |
| Gender | Male | 22 (58) |
|  | Female | 16 (42) |
| Operation | Lobectomy | 31 (82) |
|  | Pneumonectomy | 7 (18) |
| Smoking status | Former/current | 18 (53) |
|  | Never | 20 (47) |
| Histology | Adenocarcinoma | 18 (53) |
|  | Squamous cell carcinoma | 20 (47) |
| pT | 1 | 21(55) |
|  | 2 | 11(29) |
|  | 3 | 4(11) |
|  | 4 | 2(5) |
| pStage | IIIA | 32(84) |
|  | IIIB | 6(16) |

**Figure Legends**

Figure **1**. Multiplex immunofluorescence staining and multispectral imaging to visualize the expression of CD3, CD8, PD-L1 and cytokeratin (CK) in primary tumors (PTs) and metastatic lymph nodes (mLNs). (a) Fields of view (FOVs) of the center of tumor (CT) and invasive margin (IM) regions were identified at low magnification (x 10). (b) Images of representative FOVs that categorized as CT in sections with tumor tissue being too small. (c-d) FOVs of CT region in PT (c) and mLN (d). Staining was performed for CD3 for T lymphocytes (yellow), CD8 for cytotoxic T cells (green), type II CK for epithelial cells (purple), 4'-6'-diamidino-2-phenylindole (DPAI) for nuclei (blue), and PD-L1 (red). (e-f) FOVs of IM region in PT (e) and mLN (f). Hand-drawn training regions allowed images to be segmented into tumor compartment (presence of cytokeratin positivity [red]) and stroma compartment (absence of cytokeratin positivity [green]). (g-h) Densities of CD3+ and CD8+ tumor infiltrating lymphocytes (TILs) in tumor and stroma compartment of PT (g) and mLN (h) were assessed. Higher densities of CD3+ and CD8+ were observed in stroma compartment compared to tumor compartment in both PT and mLN. Significance was evaluated using a Wilcoxon signed-rank test. **** *P* < 0.0001.


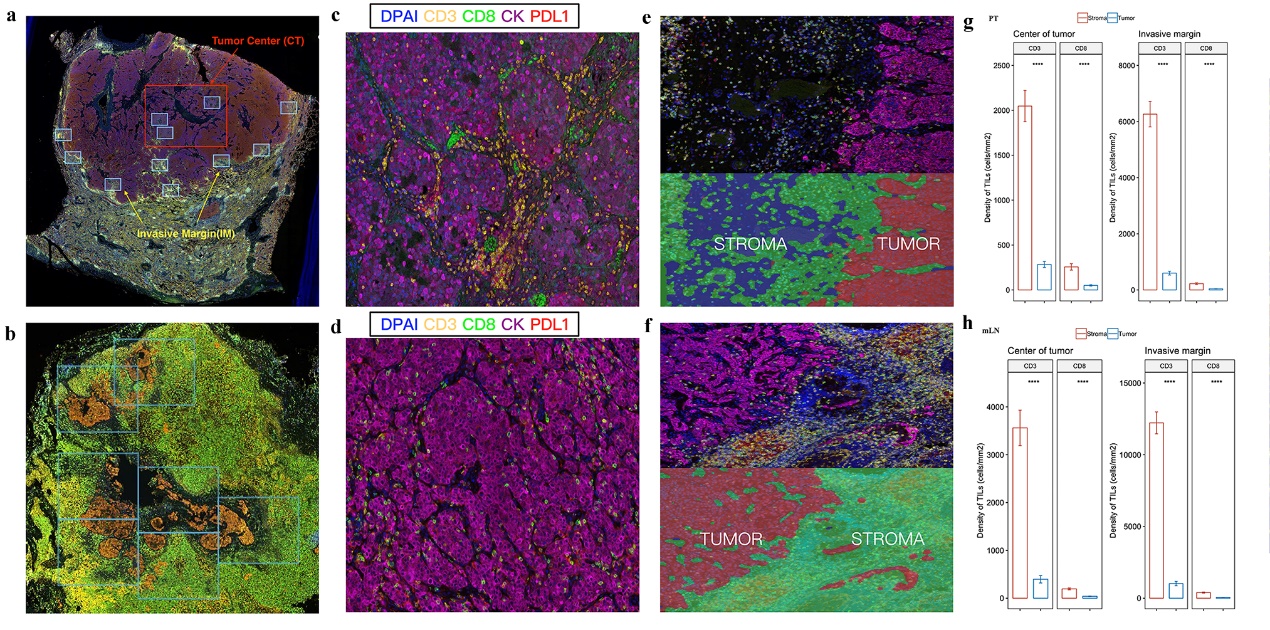


**Figure 2.** The *in situ* immune pattern of T cells and cytotoxic T cells were different in primary tumors (PTs) and metastatic lymph nodes (mLNs). (a-d) The densities of T lymphocytes (CD3+) and cytotoxic T cells (CD8+) were significantly correlated between PTs and mLNs at the center of tumor (CT) and invasive margin (IM) regions. (a) The densities of stromal T lymphocytes (S-CD3+) were significantly higher in mLNs than in PTs at both the CT and IM regions. (b, d) In tumor compartment, the densities of T cells (T-CD3+) and cytotoxic T cells (T-CD8+) were not significantly different. (c) The densities of stromal cytotoxic T cells (S-CD8+) were significantly higher in mLNs than in PTs at IM region. S = stroma; T = tumor; ns = not significant; **** *P* < 0.0001.





**Figure 3**. The densities of stromal PD-L1-positive T cells (S-PD-L1+CD3+) in the center of tumor (CT) region (a) were significantly correlated between primary tumor (PT) and metastatic lymph node (mLN); (b) in contrast, a marginally significant correlation was found in the invasive margin (IM) region. The densities and frequencies of S-PD-L1+CD3+ were significantly higher in mLNs than in PTs irrespective of location. CT = center of tumor; IM = invasive margin; S = stroma * *P* < 0.05; ** *P* < 0.01.





**Figure 4**. (a) Positron-emission tomography-computed tomography (PET/CT) imaging of the chest of a 46-year-old male patient with stage IIIA squamous cell lung cancer before and after the administration of 3 cycles of cisplatin/albumin-bound paclitaxel plus pembrolizumab. The pretreatment scan showed a primary tumor (PT) (3.3*3.6*6.8 cm; SUV max = 11.0) in left upper lobe and enlarged subaortic lymph nodes (LN) (1.9*2.9 cm; SUV max = 6.4) (upper arrow). A scan performed before surgery showed a decrease in size and FDG uptake of both PT (2.3*2.8 cm; SUV max = 3.5) and LN (1.3*2.4 cm; SUV max = 3.7) (lower arrow). (b, c) Multiplex immunofluorescence staining and fields of view (FOVs) selection of the primary tumor and subaortic lymph nodes. (d-f) Densities of stromal T cells (S-CD3+), cytotoxic T cells (S-CD8+) and PD-L1positive T cells (S-PD-L1+CD3+) were assessed in LN and PT in two regions (center of tumor [CT] and invasive margin [IM]). * *P* < 0.05; ns; not significant.

**
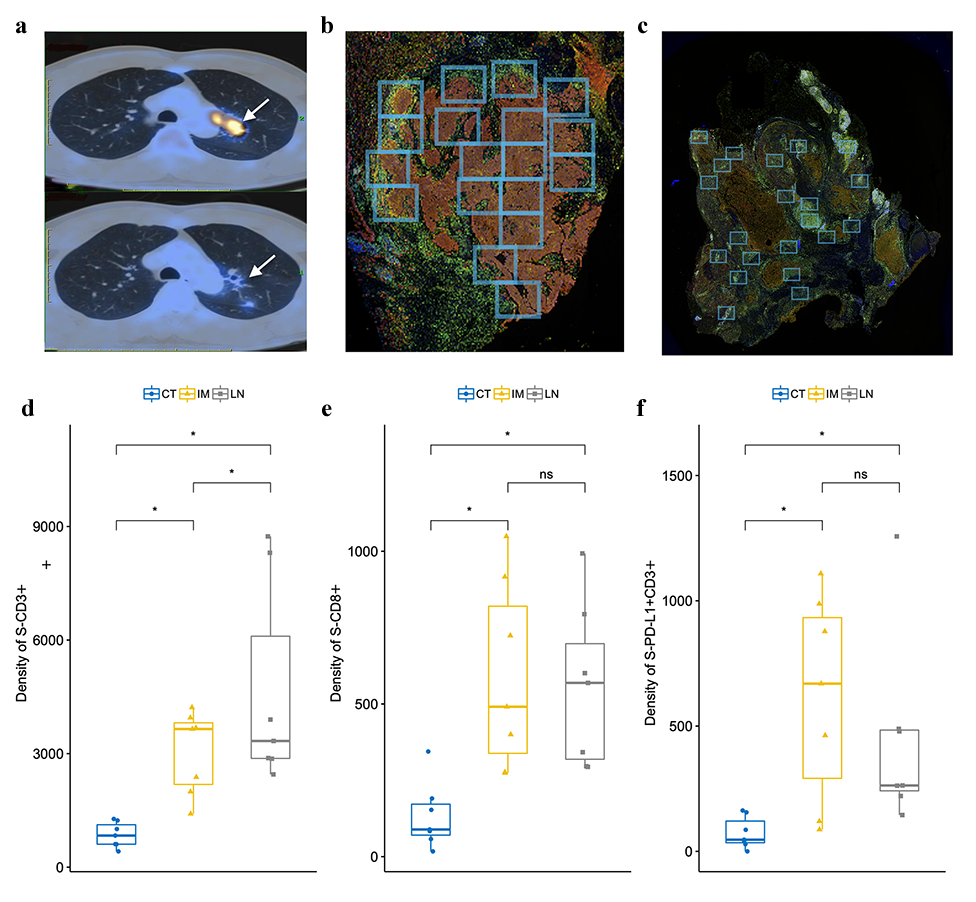
**

**Supplementary materials**

**Supplementary Table 1**. Densities and correlations of CD3+ and CD8+ lymphocyte in tumor center and invasive margin between primary tumor and metastatic lymph node

**Supplementary Table 2**. Density and frequency of S-PD-L1 positive T cells in primary tumor and metastatic lymph node

**Supplementary Table 3**. Densities of stromal CD3+, CD8+ and PD-L1-positive CD3+ lymphocytes in primary tumor and metastatic lymph nodes

**Supplementary Figure 1**. The tumor proportion score (TPS, %) was significantly correlated between primary tumor (PTs) and metastatic lymph nodes (mLNs) (r = 0.40, p = 0.016) but the combined positive score (CPS) was not (r = 0.14, *P* = 0.42).

**Supplementary Figure 2**. Forest plots showing pathological complete response in primary tumor versus complete nodal clearance (ypN0) following neoadjuvant immunotherapy (data extracted from five trials).

**Supplementary material.** Case Presentation
