# Supplementary material for: Different In Situ Immune Patterns between Primary Tumor and Lymph Node in Non-Small-Cell Lung Cancer: Potential Impact on Neoadjuvant Immunotherapy
Source: J Immunol Res. 2022 Apr 28;2022:8513747. doi: 10.1155/2022/8513747 (PMC9071859; doi:10.1155/2022/8513747)
Supplement: Supplementary Materials — Supplementary Table 1: densities and correlations of CD3+ and CD8+ lymphocyte in tumor center and invasive margin between primary tumor and metastatic lymph node. Supplementary Table 2: density and frequency of S-PD-L1-positive T cells in primary tumor and metastatic lymph node. Supplementary Table 3: densities of stromal CD3+, CD8+, and PD-L1-positive CD3+ lymphocytes in primary tumor and metastatic lymph nodes. Supplementary Figure 1: the tumor proportion score (TPS, %) was significantly correlated between primary tumors (PTs) and metastatic lymph nodes (mLNs) (r = 0.40, P = 0.016), but the combined positive score (CPS) was not (r = 0.14, P = 0.42). Supplementary Figure 2: forest plots showing pathological complete response in primary tumor versus complete nodal clearance (ypN0) following neoadjuvant immunotherapy (data extracted from five trials). Supplementary Material: case presentation. [file 8513747.f1.zip › Supplementary Material.docx]

**Supplementary material**

**Case Presentation**

Forty-six-year-old gentleman

Diagnosis: A positron-emission tomography-computed tomography (PET/CT) was used for baseline tumor staging, which showed a left upper lung (LUL) mass adjoining the pleura and extending to the left hilar (3.3*3.6*6.8 cm; SUV_max_ = 11.0). In addition, PET/CT scan showed enlarged subaortic lymph nodes with focally increased FDG uptake (1.9*2.9 cm; SUV_max_ = 6.4) with no evidence of distant metastatic disease.

A CT-guided biopsy of the primary tumor revealed poorly differentiated squamous cell lung cancer (SCC).

The patient’s tumor was staged as cT3N2M0 IIIB SCC and received induction chemotherapy/immunotherapy with 3 cycles of cisplatin/albumin-bound paclitaxel plus pembrolizumab.

Follow-up: Four weeks after the induction therapy, PET/CT scan showed a decrease in the size and FDG uptake of primary tumor (PT) with necrosis and cavity formation (2.3*2.8 cm; SUV_max_ = 3.5), and a decrease in the size of the subaortic lymph nodes (1.3*2.4 cm; SUV_max_ = 3.7) with no distant metastases.

According to the Response Evaluation Criteria in Solid Tumors (RECIST), version 1.1, the tumor achieved partial response (PR). Left upper lobe lobectomy and systemic lymphadenectomy was performed 6 days following the second PET/CT scan.

Pathologic report: PT: 2*1*1cm, poorly differentiated SCC, no pleura invasion.

Absence of lymphatic vascular invasion. Lymph nodes were free of metastasis.

**Method**:

***Histopathological assessment of PT and mLN for immune-mediated pathologic response***

Immune-related pathologic response criteria (irPRC) and immune-mediated responses proposed by Cottrell, T. R *et al*^1^ were evaluated on hematoxylin and eosin (H&E)-stained slides of PT and metastatic lymph node (mLN) resection specimens. In the irPRC system, the total tumor bed is defined by residual viable tumor (RVT) + necrosis + regression bed. The total tumor bed is estimated to calculate the percentage of immune-related RVT (%irRVT) (viable tumor area/total tumor bed area * 100). The immune-mediated responses evaluated include: Presence or absence of tertiary lymphoid structures (TLS), dense plasma cell infiltrate (≥100 plasma cells/high power field (HPF) in at least two HPFs), foamy (lipid-filled) macrophages, giant cells, cholesterol clefs, neovascularization, necrosis and proliferative (new) fibrosis (high fibroblast to collagen ratio).

***Results***

***mLN showed better immune-mediated pathologic response than PT after neoadjuvant chemo-immunotherapy***

PT specimens showed RVT remaining and 2*1*1 cm in size, while all lymph nodes resection specimens showed no RVT remaining. The H&E stained slides of PT and subaortic lymph nodes were assessed for immune-related pathologic response. Both PT and subaortic lymph nodes showed immune-mediated responses such as proliferative fibrosis, neovascularization and necrosis. We also observed immune-mediated responses exclusive to subaortic lymph nodes such as foamy macrophages and cholesterol clefts. TLS, giant cells and dense plasma cell infiltrates were absent in both PT and subaortic lymph nodes. %irRVT was 60% in PT and 0% in subaortic lymph nodes (pathologic complete response, pCR). Multiplex immunofluorescence staining and multispectral imaging were performed on the slides of PT and subaortic lymph nodes. FOVs of PT were taken at CT and IM respectively as described. Because no visible tumor tissue was found on the section of subaortic lymph nodes, FOVs were taken on stromal area where CD3 and CD8 aggregated in the whole section.

The densities of S-CD3 and S-CD8 at CT and IM in PT were both statistically correlated with subaortic lymph nodes. The densities of S-CD3 at CT and IM as well as S-CD8 at CT in subaortic lymph nodes were significantly higher than PT, while no difference was found in the density of S-CD8 at IM. The density of S-PDL1+CD3 in subaortic lymph nodes was significantly higher than PT at CT. Yet density of S-PDL1+CD3 in PT at IM was significantly different from subaortic lymph nodes.

**Reference**

1. Cottrell TR, Thompson ED, Forde PM, et al. Pathologic features of response to neoadjuvant anti-PD-1 in resected non-small-cell lung carcinoma: a proposal for quantitative immune-related pathologic response criteria (irPRC). *Ann Oncol*. 2018;29(8):1853-1860.
