# Supplementary material for: Different In Situ Immune Patterns between Primary Tumor and Lymph Node in Non-Small-Cell Lung Cancer: Potential Impact on Neoadjuvant Immunotherapy
Source: J Immunol Res. 2022 Apr 28;2022:8513747. doi: 10.1155/2022/8513747 (PMC9071859; doi:10.1155/2022/8513747)
Supplement: Supplementary Materials — Supplementary Table 1: densities and correlations of CD3+ and CD8+ lymphocyte in tumor center and invasive margin between primary tumor and metastatic lymph node. Supplementary Table 2: density and frequency of S-PD-L1-positive T cells in primary tumor and metastatic lymph node. Supplementary Table 3: densities of stromal CD3+, CD8+, and PD-L1-positive CD3+ lymphocytes in primary tumor and metastatic lymph nodes. Supplementary Figure 1: the tumor proportion score (TPS, %) was significantly correlated between primary tumors (PTs) and metastatic lymph nodes (mLNs) (r = 0.40, P = 0.016), but the combined positive score (CPS) was not (r = 0.14, P = 0.42). Supplementary Figure 2: forest plots showing pathological complete response in primary tumor versus complete nodal clearance (ypN0) following neoadjuvant immunotherapy (data extracted from five trials). Supplementary Material: case presentation. [file 8513747.f1.zip › Supplementary Tables.docx]

**Supplementary Table 1**. Densities and correlations of CD3+ and CD8+ lymphocyte in tumor center and invasive margin between primary tumor and metastatic lymph node

| Markers | Median Cell density(cells/mm^2^) | | P^†^ | r^‡^ | *P*^‡^ |
| --- | --- | --- | --- | --- | --- |
|  | PT | mLN |  |  |  |
| CT  S-CD3+  S-CD8+  T-CD3+  T-CD8+ | 1228.80  92.88  91.37  12.21 | 1458.0  102.78  112.68  8.03 | <0.0001  0.105  0.362  0.184 | 0.61  0.50  0.60  0.50 | <0.0001  <0.0001  <0.0001  <0.0001 |
| IM  S-CD3+  S-CD8+  T-CD3+  T-CD8+ | 4219.30  74.86  240.40  11.51 | 8479.00  198.84  249.30  10.02 | <0.0001  <0.0001  0.362  0.469 | 0.72  0.49  0.63  0.64 | <0.0001  <0.0001  <0.0001  <0.0001 |

^†^ Wilcoxon signed-rank test; ^‡^ Spearman rank correlation coefficient

Abbreviation: PT = primary tumor; mLN = metastatic lymph node; CT = center of tumor; IM = invasive margin; S = stroma; T = tumor.

**Supplementary Table 2**. Density and frequency of S-PD-L1 positive T cells in primary tumor and metastatic lymph node

| Median | PT | mLN | *P*^†^ | r^‡^ | *P*^‡^ |
| --- | --- | --- | --- | --- | --- |
| CT  S-PD-L1+CD3+  (cells/mm^2^)  % PD-L1+CD3+ | 60.43  2.97 | 146.80  5.26 | 0.017  0.006 | 0.39  0.30 | 0.020  0.085 |
| IM  S-PD-L1+CD3+  (cells/mm^2^)  % PD-L1+CD3+ | 178.14  4.49 | 1151.10  14.64 | 0.001  0.003 | 0.35  0.24 | 0.051  0.196 |

^†^ Wilcoxon signed-rank test; ^‡^ Spearman rank correlation coefficient

Abbreviation: PT = primary tumor; mLN = metastatic lymph node; CT = center of tumor; IM = invasive margin; S = stroma.

**Supplementary Table 3**. Densities of stromal CD3+, CD8+ and PD-L1-positive CD3+ lymphocytes in primary tumor and metastatic lymph nodes.

| Markers | Median cell density (cells/mm2) | | | P ^†^ | P ^‡^ | P ^§^ |
| --- | --- | --- | --- | --- | --- | --- |
|  | PT (CT) | PT (IM) | mLNs |  |  |  |
| S-CD3+  S-CD8+  S-PDL1+CD3+ | 834.98  88.97  46.46 | 3649.78  490.80  669.28 | 3334.00  569.00  263.00 | 0.016  0.016  0.016 | 0.047  0.375  0.219 | 0.016  0.016  0.016 |

Wilcoxon signed-rank test: ^†^ PT (CT) and mLNs; ^‡^ PT (IM) and mLNs; ^§^ PT (CT) and PT (IM).

Abbreviation: PT = primary tumor; mLNs = metastatic lymph nodes; CT = center of tumor; IM = invasive margin; S = stroma.
