# Supplementary figures and images for: Different In Situ Immune Patterns between Primary Tumor and Lymph Node in Non-Small-Cell Lung Cancer: Potential Impact on Neoadjuvant Immunotherapy
Source: J Immunol Res. 2022 Apr 28;2022:8513747. doi: 10.1155/2022/8513747 (PMC9071859; doi:10.1155/2022/8513747)

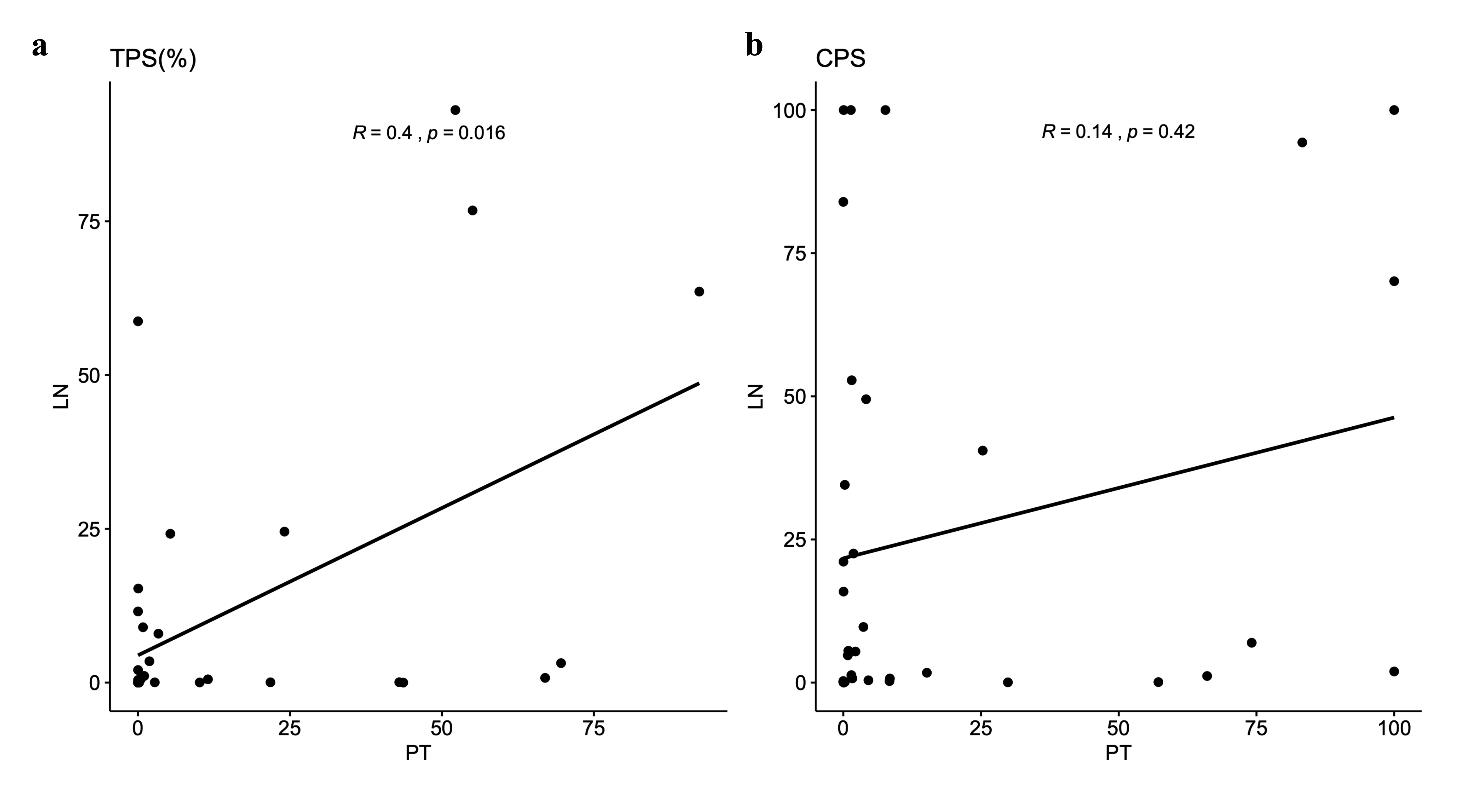

Supplement: Supplementary Materials — Supplementary Table 1: densities and correlations of CD3+ and CD8+ lymphocyte in tumor center and invasive margin between primary tumor and metastatic lymph node. Supplementary Table 2: density and frequency of S-PD-L1-positive T cells in primary tumor and metastatic lymph node. Supplementary Table 3: densities of stromal CD3+, CD8+, and PD-L1-positive CD3+ lymphocytes in primary tumor and metastatic lymph nodes. Supplementary Figure 1: the tumor proportion score (TPS, %) was significantly correlated between primary tumors (PTs) and metastatic lymph nodes (mLNs) (r = 0.40, P = 0.016), but the combined positive score (CPS) was not (r = 0.14, P = 0.42). Supplementary Figure 2: forest plots showing pathological complete response in primary tumor versus complete nodal clearance (ypN0) following neoadjuvant immunotherapy (data extracted from five trials). Supplementary Material: case presentation. [file 8513747.f1.zip › Supplementary Fig1.jpg]

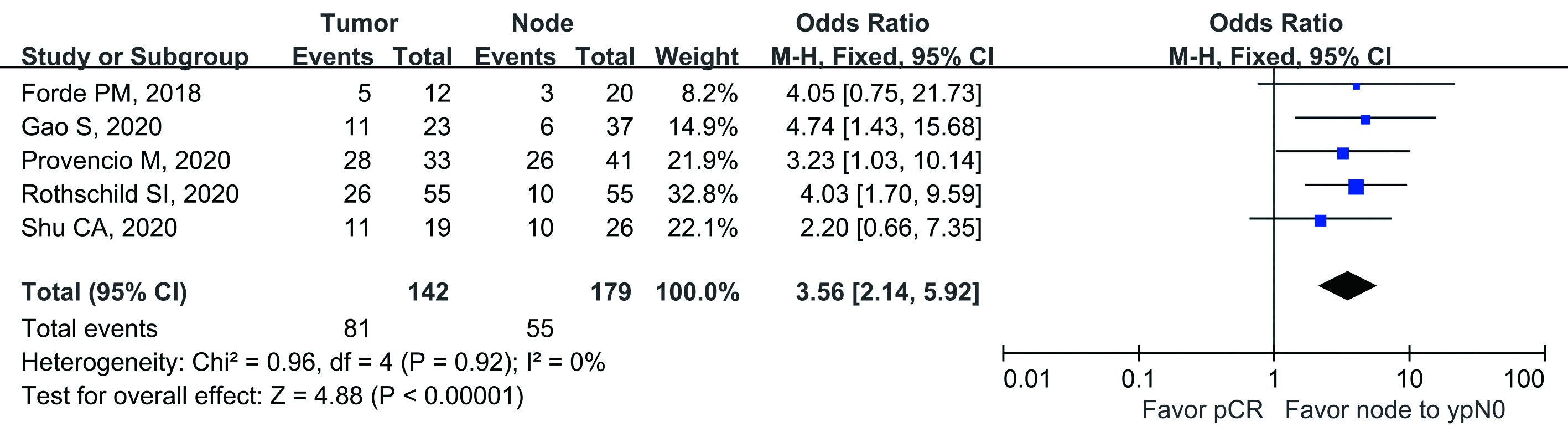

Supplement: Supplementary Materials — Supplementary Table 1: densities and correlations of CD3+ and CD8+ lymphocyte in tumor center and invasive margin between primary tumor and metastatic lymph node. Supplementary Table 2: density and frequency of S-PD-L1-positive T cells in primary tumor and metastatic lymph node. Supplementary Table 3: densities of stromal CD3+, CD8+, and PD-L1-positive CD3+ lymphocytes in primary tumor and metastatic lymph nodes. Supplementary Figure 1: the tumor proportion score (TPS, %) was significantly correlated between primary tumors (PTs) and metastatic lymph nodes (mLNs) (r = 0.40, P = 0.016), but the combined positive score (CPS) was not (r = 0.14, P = 0.42). Supplementary Figure 2: forest plots showing pathological complete response in primary tumor versus complete nodal clearance (ypN0) following neoadjuvant immunotherapy (data extracted from five trials). Supplementary Material: case presentation. [file 8513747.f1.zip › Supplementary Fig2.jpg]
